# Pediatrics

**Published:** 1896-06

**Authors:** Maud J. Frye

**Affiliations:** Clinical instructor in diseases of children, Medical Department, University of Buffalo


### PEDIATRICS.

Reported by MAUD J. FRYE, M. D.,
Clinical instructor in diseases of children, Medical Department, University of Buffalo.

#### YEAST NUCLEIN IN THE TREATMENT OF HIP-JOINT DISEASE.

Hitchcock, of Detroit, (*American Lancet*, abstracted in *N. Y. Med. Jour.*,) reports a case of hip-joint disease in which great improvement and apparent cure followed the use of yeast nuclein. The remedy was used hypodermically, and the site chosen for injection was the region immediately around the affected joint. The first few injections were made daily, afterwards on alternate days. In the beginning twelve minims only were used ; later the amount was increased to fifty. The reaction was moderate—at times some pain and a burning sensation about the site of injection and a moderate rise of temperature. To be of avail the nuclein must be used early. Dr. Hitchcock believes the value of nuclein to lie in its germicidal powers. The preparation used by him was the one-per-cent. solution manufactured by Parke, Davis & Co.

---

#### RENAL COLIC IN INFANCY.

Gibbons (*British Medical Journal*, January 18, 1896,) describes the occurrence of renal colic in infancy. In none of the patients was a distinct calculus found, but there was an abundance of free uric acid and small masses of mortar-like material, consisting of uric acid, were passed. The attacks occurred in children of gouty parents, and presented all the signs and symptoms of acute renal colic. Dr. Guthrie, discussing the paper, stated that he had seen such cases among the poor, generally in subjects of a rheumatic diathesis.

---

### TREATMENT OF PNEUMONIA IN CHILDREN.

*Archives of Pediatrics* devotes considerable space in the April number to this subject. Drs. Geo. M. Swift, L. Emmet Holt and W. P. Northrup, of New York; Dr. J. P. Crozer Griffith, of Philadelphia; Dr. E. M. Buckingham, of Boston, and Dr. Samuel S. Adams, of Washington, contribute articles giving briefly the treatment followed in the various children's hospitals which they attend. The treatment on the whole is strikingly uniform. The following may be taken as representing the average:

1. In all cases attention is given to hygiene—warm, airy, well-ventilated rooms, careful attention to regulation of nutrition and digestion.

2. For the relief of pain, counter-irritation, opium if needed.

3. For cough, inhalations; in some hospitals the croup tent; opium if needed. So-called expectorants, except chloride of ammonia, are almost entirely discarded.

4. Fever *per se* is not considered as requiring treatment. If the nervous symptoms demand, antipyretic measures are used, preferably hydrotherapy, the means employed being sponge baths, warm or cold, tub baths, the cold pack and ice bags. Antipyretic drugs are employed by some.

5. Stimulants are used as indication arises. Those to which all give prominence are alcohol in the form of whisky or brandy, strychnine, which children bear well, and nitroglycerin, in some cases extremely valuable. Stimulants may be given hypodermatically.

6. So far as specific remedies are concerned, but one is suggested, the chloride of calcium, which is given by Dr. George M. Swift in lobar pneumonia in from two to five-grain doses every two or three hours.

---

### TYPHOID FEVER IN YOUNG CHILDREN.

NORTHRUP (*Archives of Pediatrics*, January, 1896,) contributes an article tending to corroborate the general belief that children under two are markedly insusceptible to typhoid. The epidemic at Stamford, Conn., in the spring of 1895, was due to a contaminated milk supply. In all, 406 cases occurred. Only four of these were in children under 2 years of age. Three cases occurred between two and three. The course of the disease in these children was similar to that in adults. The characteristic eruption is a valuable aid to diagnosis. The author remarks that he desires to encourage a healthy scepticism as to typhoid in an infant in the absence of an epidemic, if the case lacks the classic symptoms and signs which would lead to a diagnosis in an adult. Autopsy findings, as shown by Dr. Northrup, are misleading ; swelling of Peyer's patches and the solitary follicles are common in simple intestinal disease.

